# Clinical and bacterial determinants of unfavorable tuberculosis treatment outcomes: an observational study in Georgia

**DOI:** 10.1186/s13073-025-01555-0

**Published:** 2025-11-14

**Authors:** Galo A. Goig, Chloé Loiseau, Nino Maghradze, Kakha Mchedlishvili, Teona Avaliani, Ana Tsutsunava, Daniela Brites, Sevda Kalkan, Sonia Borrell, Rusudan Aspindzelashvili, Zaza Avaliani, Maia Kipiani, Nestani Tukvadze, Levan Jugheli, Sebastien Gagneux

**Affiliations:** 1 Swiss Tropical and Public Health Institute, Kreuzstrasse 2, Allschwil, 4123 Switzerland; 2 University of Basel, Basel, Switzerland; 3 National Center for Tuberculosis and Lung Diseases (NCTLD), Tbilisi, Georgia; 4 European University, Tbilisi, Georgia; 5 David Tvildiani Medical University (DTMU), Tbilisi, Georgia; 6 The University of Georgia, Tbilisi, Georgia

**Keywords:** Tuberculosis, Treatment outcomes, Genomics, GWAS, Precision medicine, Antimicrobial resistance

## Abstract

**Background:**

Tuberculosis (TB) remains a major public health concern. Improving TB control programs and treatment success requires a deeper understanding of the factors that determine disease presentation and treatment outcomes. While the importance of patient factors is well established, our understanding of the bacterial determinants of disease presentation and treatment outcomes in TB remains limited.

**Methods:**

In this study, we analyzed the *Mycobacterium tuberculosis* complex (MTBC) genomes and the associated clinical data from 4529 TB patients in the country of Georgia covering a period of 13 years. We used multivariable modeling together with genome-wide association studies (GWAS) to identify patient and bacterial factors that determine TB disease manifestation and clinical outcomes.

**Results:**

Multivariable modelling confirmed the role of demographic and clinical factors in determining treatment outcomes, as well as the efficacy of novel TB treatments containing bedaquiline. In addition, we found that several bacterial factors, including the MTBC lineage, the specific mutations conferring resistance to rifampicin and fluoroquinolones, as well as a high bacterial burden, were associated with unfavorable outcomes. GWAS analyses revealed no bacterial genetic mutations associated with treatment outcomes beyond the known drug resistance-conferring mutations. However, we found that mutations in the bacterial gene *sufD* were linked to a reduced risk of lung cavities and a lower bacterial burden within patients. By contrast, specific mutations conferring resistance to rifampicin and fitness compensatory mutations were associated with a higher bacterial burden.

**Conclusions:**

Our results show that both patient and bacterial factors determine disease presentation and clinical outcomes in TB. They also support the rationale of optimizing treatment regimens against drug-resistant TB with existing drugs based on the specific genetic features of the pathogen. Finally, our results highlight *sufD* as a possible therapeutic candidate.

**Supplementary Information:**

The online version contains supplementary material available at 10.1186/s13073-025-01555-0.

## Background

Despite being preventable and treatable, tuberculosis (TB) remains one of the top ten leading causes of human mortality worldwide, and the leading cause of death from a single infectious agent [1]. For drug-susceptible TB, treatment success rates are high; yet globally, more than 12% of TB patients fail treatment [1]. The situation is considerably worse for drug-resistant forms of TB, with success rates averaging only 68% for multidrug-resistant TB (MDR-TB) and 44% for extensively drug-resistant TB (XDR-TB) [1, 2]. Recently, the World Health Organization (WHO) endorsed the use of regimens based on the novel and repurposed drugs bedaquiline, pretomanid, and linezolid, with or without moxifloxacin (BpaL(M)), that have improved treatment outcomes for MDR-TB [3]. However, despite these BPaL(M) regimens being introduced only recently, resistance to these drugs is already emerging, and strains resistant to BPaL(M) are spreading between patients [4]. In order to preserve new regimens and improve TB control programs, we need to better understand the factors that influence treatment outcomes of TB patients across epidemiological settings.

The clinical and demographic factors that influence treatment outcomes are generally well understood. Comorbidities such as HIV, diabetes, or alcohol abuse; patient characteristics such as age, sex, or body mass index (BMI); and social factors such as unemployment, imprisonment, or homelessness are well-documented determinants of TB outcomes (refer to Peetluk et al*.* for a systematic review on modeling studies [5]). By contrast, factors related to the *Mycobacterium tuberculosis* complex (MTBC), which is the causative agent of TB, are less understood. A key bacterial factor is antibiotic resistance. It is well established that resistance to TB drugs leads to unfavorable treatment outcomes [1]. However, drug resistance is not a binary trait but a spectrum that ranges from full susceptibility to varying degrees of resistance. The level of drug resistance itself is influenced by the specific resistance mutations conferring resistance to the particular drug [6], as well as by the bacterial genetic background in which the mutation occurs [7, 8]. Therefore, genetic analyses of the infecting MTBC strains may provide opportunities to optimize treatments and improve treatment success rates. For example, several studies have shown that mutations associated with a high level of resistance to fluoroquinolones were also associated with unfavorable patient treatment outcomes [9, 10]. However, these associations have only been studied for prevalent drug resistance-conferring mutations, and often without controlling for key patient factors. Therefore, our understanding of how the different resistance-conferring mutations may influence treatment outcomes in TB remains limited.

Other bacterial factors could also play a role in determining TB disease manifestation and treatment outcomes. For example, “modern Beijing” strains have been associated with unfavorable treatment outcomes [11], while strains belonging to MTBC Lineage 1 have been associated with disseminated TB and bone disease [12]. However, only a few studies have attempted to find novel bacterial determinants of TB treatment outcomes [7, 13–16], and most of these did not take into account other relevant clinical and demographic factors or were limited by a small sample size. As a result, our understanding of the overall clinical relevance of bacterial genetics in determining treatment outcomes and disease progression in TB remains limited.

In this study, we analyzed 4529 MTBC genomes together with the associated clinical data from patients diagnosed with TB in Georgia over a period of 13 years. By integrating both bacterial and patient data, we aimed to identify bacterial and host factors that are relevant to patient treatment outcomes and disease manifestation in TB.

## Methods

### Study cohort and samples analyzed

Between 1st October 2010 and 31st December 2023, *M. tuberculosis* isolates from all bacteriologically confirmed MDR-TB cases were collected and cultured at the National Center for Tuberculosis and Lung Disease in Tbilisi (NCTLD), Georgia. Additionally, a separate subset of *M. tuberculosis* isolates from all TB cases (including drug-susceptible cases) was collected between the years 2014–2016 (Additional File 1: Fig S1). All isolates were derived from routine diagnostic sputum samples. These samples were collected at the NCTLD, homogenized, and decontaminated with NaOH. Following decontamination, they were inoculated into the BACTEC MGIT 960 system for primary culture. All positive MGIT isolates were then subcultured onto Middlebrook 7H10 solid media plates for DNA extraction. DNA was extracted using a phenol-chloroform extraction and subsequently sent for whole-genome sequencing (WGS) on Illumina NovaSeq 6000 and Illumina HiSeq 2500 platforms at the genomics facility of the University of Basel and the Department of Biosystems Science and Engineering at ETHZ in Basel, Switzerland. Pseudo-anonymized patient-related data were routinely collected at NCTLD. In this study, we specifically aimed to evaluate potential associations of bacterial factors with treatment outcomes; consequently, patients whose treatment outcome was classified as “lost to follow-up” were excluded. Treatment outcomes were defined following the standard definitions of the WHO [17]: favorable for cases that were cured or that completed treatment, and unfavorable for those who showed treatment failure or died.

### Whole-genome sequencing analysis

WGS data was analyzed as previously described [4]. Briefly, sequencing data were processed to remove low-quality sequences and reads originating from non-MTBC organisms. Additionally, samples with an average sequencing depth below 20× or with more than 10% of non-MTBC DNA were discarded. Mapping and variant calling were performed using the inferred chromosome of the MTBC ancestor as the reference genome. Variants called in repetitive regions such as PE, PPE, and PGRS genes or phages were discarded. To identify drug resistance-conferring mutations, we classified any mutation with at least 10% frequency following the criteria established in the second edition of the WHO catalog [18]. We considered isolates to be pre-extensively drug-resistant (pre-XDR) if they showed resistance to rifampicin, isoniazid, and any fluoroquinolone. We considered isolates to be extensively drug-resistant (XDR) if they showed a pre-XDR genotype and additional resistance to one of the following: bedaquiline, delamanid, or linezolid. Estimated minimum inhibitory concentrations (MIC) for each drug were assigned based on the results of two modeling studies of data derived from the CRyPTIC Consortium [6, 19] (Additional File 1: Supplementary Methods).

To infer a phylogeny, we created an alignment with all non-redundant polymorphic positions in the dataset by concatenating all high-quality SNPs, excluding positions associated with drug resistance. SNPs with an allele frequency lower than 90% or positions covered with less than 7 reads were encoded with an “X”. Based on this alignment, we built a maximum-likelihood phylogeny using IQ-TREE 2 [20] with the general time-reversible model of sequence evolution, 1000 bootstrap replicates, and indicating the number of invariant sites of each nucleotide.

### Statistical analysis and modeling

The data, a detailed step-by-step description of all analyses performed in this study, as well as supplementary results, tables, and figures, are available in a supplementary public repository [21]. We provide this as supplementary material and refer to it throughout the manuscript, alongside an additional file with supplementary methods and results. Briefly, to select the variables to be included in the final logistic regression models, we initially considered 66 available clinical and bacterial variables potentially relevant for patient treatment outcomes. Missing data for categorical variables were encoded as “missing”, whereas continuous data for BMI and time to culture positivity (TTP) were imputed using multivariate imputation by chained equations (MICE), specifying random forest as the imputation model (Supplementary Repository)*.* To select for those variables that better explain patient treatment outcomes, we performed feature selection based on two methods. First, we conducted a least absolute shrinkage and selection operator (LASSO) regularization analysis. Second, we built a random forest model. We initially included in the model the combination of the predictors whose coefficients were not shrunk to zero in the LASSO regularization, and the top ten predictors of the random forest (Additional File 1: Supplementary Methods). Model performances were assessed by tenfold cross-validation using the area under the precision-recall curve, given the high-class imbalance of our data (10.9% of the positive class, unfavorable outcome).

### Genome-wide association studies

To find bacterial mutations associated with treatment outcomes and disease manifestation, we used the linear mixed effect model of pyseer [22]. The mutations tested only included single-nucleotide polymorphisms and small indels, and therefore large structural variants were not considered. The GWAS analyses were adjusted by diagnosis date, patient age, sex, diabetes, HIV-coinfection, employment, alcohol intake, drug abuse, treatment for other diseases, and drug resistance profile, as well as by the bacterial population structure. To correct for the population structure, we calculated a similarity matrix with pyseer. Additionally, we carried out gene burden tests. GWAS *p*-values were adjusted using the Bonferroni correction.

### Ethics approval and consent to participate

The institutional Review Board of the NCTLD in Tbilisi, Georgia and the Ethics Commission of North- and Central Switzerland granted ethical approval for this study. The ethics committees waived the need for individual patient consent since only anonymized clinical data were used.

## Results

### Description of the study cohort

**Fig. 1:**
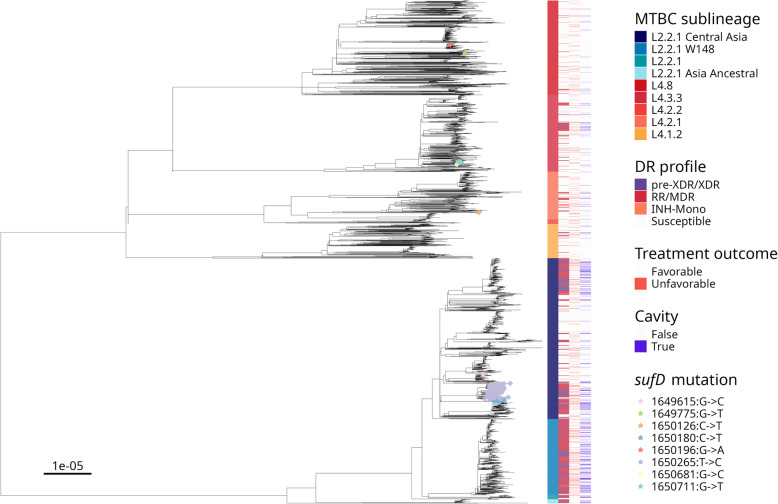
Phylogeny of 3399 MTBC isolates from TB patients with favorable or unfavorable treatment outcomes. The tree scale indicates substitutions per site. Mixed infections with different sublineages and minority sublineages were excluded for clarity. In the figure, the susceptible category of the drug resistance (DR) profile includes strains resistant to drugs other than isoniazid and rifampicin. Isolates carrying different non-synonymous mutations in *Rv1462* (*sufD*) are indicated with a colored star. Notably, 57 strains carrying *sufD* mutations were excluded from the figure as their patient outcomes were lost to follow-up

Out of 4368 TB patients with a high-quality MTBC genome (96.4%), 3579 (81.9%) had known treatment outcomes, 389 of which (10.9%) were unfavorable (Additional File 1: Fig S1). The median patient age was 39 years, and 3144 patients (72.0%) were male. The HIV-coinfection status was negative for 3544 patients (81%), positive for 145 patients (3%), and unknown for 679 patients (16%). Based on our genomic analysis, 2,375 MTBC isolates (54.4%) were susceptible to all drugs, 1338 (30.6%) were MDR, 466 (10.7%) were pre-XDR, and 23 (0.5%) were XDR. Among drug-resistant cases, 1579 (79.2%) were treated with fluoroquinolones, and 647 (32.5%) were treated with regimens containing bedaquiline, delamanid, and/or linezolid. A complete description of the dataset, including the distribution of MTBC lineages (Fig. 1), drug resistance-conferring mutations (Additional File 1: Fig S2), as well as the study population, is available in the Supplementary Repository.

### Clinical and bacterial factors associated with TB treatment outcomes

To identify factors associated with treatment outcomes, we analyzed all patients with available treatment outcome data, excluding those lost to follow-up (*n *= 3579; Additional File 1: Fig S1). Out of the 66 variables initially considered, 13 were included in the final model, which we refer to as the “base model” (Table 1; Additional File 1: Fig S3). The model performance assessed by tenfold cross-validation showed an area under the receiver operating characteristic curve of 0.81 and an area under the precision-recall curve of 0.44 (0.11 for a random classifier). These metrics show that the model captures a substantial part of the signal explaining treatment outcomes. In the base model, previous treatment failure, defaulting treatment, recurrent TB, being unemployed, male sex, older age, lower BMI, presenting with dyspnea, HIV-coinfection, being treated for other diseases, any form of drug resistance, and a shorter TTP (i.e., higher bacterial burden) were associated with unfavorable outcomes, while being treated with regimens containing bedaquiline, delamanid and/or linezolid was strongly associated with favorable outcomes (Table 1). In general, patients with missing data for employment, drug abuse, dyspnea, and HIV status were more likely to present unfavorable outcomes.

**Table 1:** Variables associated with unfavorable treatment outcomes in a multivariable logistic regression. We refer to this model as the “base” model)

Variable	aOR	95% CI	*p*-value
Patient age (years)	1.05	1.04, 1.06	**< 0.001**
Time to culture positivity (days)	0.95	0.92, 0.98	**0.001**
BMI (kg/m^2^)	0.95	0.81, 1	**0.037**
Diagnosis date	1.04	0.98, 1.10	0.15
Case definition: previous treatment failure	4.47	2.09, 9.40	**< 0.001**
Case definition: other	1.49	0.94, 2.33	0.083
Case definition: recurrent TB	1.84	1.30, 2.58	**< 0.001**
Case definition: treatment after defaulting	4.84	3.16, 7.37	**< 0.001**
INH monoresistant TB	3.31	1.40, 7.18	**0.004**
RR/MDR-TB	8.48	4.98, 14.50	**< 0.001**
pre-XDR/XDR-TB	24.2	13.5, 44.0	**< 0.001**
Male	1.63	1.20, 2.24	**0.002**
Work status: missing	3.52	1.86, 6.82	**< 0.001**
Work status: unemployed	2.28	1.42, 3.87	**0.001**
HIV-coinfection: missing	1.48	1.06, 2.04	**0.02**
HIV-coinfection: yes	1.72	0.95, 3.04	0.068
Drug abuse: missing	2.99	1.94, 4.59	**< 0.001**
Drug abuse: yes	0.64	0.19, 1.82	0.4
Treatment for other diseases: missing	0.89	0.48, 1.58	0.7
Treatment for other diseases: yes	1.69	1.10, 2.60	**0.017**
Dyspnea: missing data	2.50	1.22, 5.06	**0.012**
Dyspnea: yes	2.31	1.49, 3.56	**< 0.001**
Regimen containing Bdq/Dlm/Lzd	0.22	0.14, 0.37	**< 0.001**

*Abbreviations:* *BMI* Body mass index, *INH* Isoniazid, *TB* Tuberculosis, *RR/MDR *Rifampicin-resistant/multidrug-resistant, *XDR* Extensively drug-resistant, *Bdq* Bedaquiline, *Dlm* Delamanid, *Lzd* Linezolid, *aOR* Adjusted odds ratio, *CI* Confidence interval

### The association of MTBC lineage with TB treatment outcomes

We tested whether MTBC lineage was associated with treatment outcomes by adding lineage as a predictor to the base model (Additional File 1). In this model, lineage was not a relevant predictor. However, because a previous meta-analysis found that “Beijing” strains (L2.2.1) were associated with unfavorable treatment outcomes only among drug-susceptible cases [11], and because most L2 strains in Georgia belong to L2.2.1, we sought to replicate these results in our study. In support of these previous results, we found that L2 showed higher odds of unfavorable outcomes as compared to L4 among drug-susceptible cases (L2 adjusted odds ratio (aOR) = 1.51; 95% confidence interval (CI) = 1.02–2.19; *p*-value = 0.035; *n* = 2153), but not among drug-resistant cases (aOR = 0.95; CI = 0.60–1.52; *p*-value = 0.8; Supplementary Repository, Additional File 1: Table S1). Additionally, we tested whether different MTBC sublineages may be associated with treatment outcomes but found no evidence for this (Supplementary Repository).

### The association of mixed infections with TB treatment outcomes

In the analysis incorporating MTBC lineages, we observed that mixed infections (*n* = 53) were not associated with unfavorable outcomes (aOR = 2.81; CI = 0.42–11.0; *p*-value = 0.2; Supplementary Repository; Additional File 1: Table S1). However, in our initial models, mixed infections were defined only based on the coexistence of different sublineages, a definition that does not consider infections caused by different MTBC genotypes belonging to the same sublineage. To address this, we used the ratio of unfixed to fixed SNPs as a measure of genetic diversity, which increases in isolates harboring multiple genotypes. When measured by this ratio, the genetic diversity of the isolates was not associated with unfavorable outcomes in our cohort when assessed by univariate (t-test *p*-value = 0.8) and multivariate analyses (aOR = 0.95; CI = 0.76–1.10; *p*-value = 0.8; Additional File 1: Supplementary Results; Supplementary Repository).

### The effect of specific drug resistance-conferring mutations on TB treatment outcomes

Using the base model, we assessed whether specific mutations conferring resistance to different TB drugs could be differentially associated with unfavorable treatment outcomes. One problem with this analysis is that assessing the effect of many different drug resistance-conferring mutations leads to a high degree of stratification and unreliable estimates (Supplementary Repository). To circumvent this, we built alternative models in which each individual mutation was substituted by its estimated effect on the MIC based on modeling studies of data derived from the CryPTIC consortium [6, 19] (Additional File 1: Supplementary Methods). For this analysis, we excluded isolates without drug resistance-conferring mutations since no MIC can be imputed to these isolates based on genome analysis. A limitation of this analysis is that isoniazid resistance could not be modeled since isoniazid resistance was dominated by a single mutation (KatG Ser315Thr, Additional File 1: Fig S2). Similarly, pyrazinamide resistance could not be modeled as MIC values for pyrazinamide resistance-conferring mutations are not available. Therefore, our study focused on modeling the effect of resistance to rifampicin and fluoroquinolones, since resistance to these drugs emerged as relevant predictors of unfavorable outcomes (Table 1; Supplementary Repository). When evaluating isolates with rifampicin resistance-conferring mutations (*n* = 1348), increasing rifampicin MICs were associated with unfavorable treatment outcomes (aOR = 1.27 per each log_2_(MIC) increase, CI = 1.19–1.36; *p*-value < 0.001). In the case of isolates resistant both to rifampicin and fluoroquinolones (*n* = 344), increasing MICs to rifampicin (aOR = 1.28, CI = 1.20–1.36; *p*-value < 0.001) and to fluoroquinolones (aOR = 1.36, CI = 1.23–1.51; *p*-value < 0.001) were associated with unfavorable treatment outcomes (Additional File 1: Table S2; Additional File 1: Table S3).

Additionally, we tested whether heteroresistance may be linked with treatment outcomes. Note that this analysis specifically assessed the effect of heteroresistance (coexistence of drug-susceptible and drug-resistant strains), independent of the effect of drug resistance itself. In multivariable analysis, we did not find heteroresistance to be associated with treatment outcomes (Supplementary Repository; Additional File 1: Table S4).

### GWAS of variants associated with TB treatment outcomes and disease manifestation

In our GWAS analyses, we did not find any MTBC genetic variant associated with treatment outcomes after correcting for multiple testing (Supplementary Repository). By contrast, we found that non-synonymous mutations in the gene *Rv1462* (*sufD*) were associated with lower odds of presenting with cavitary disease (aOR = 0.9; CI = 0.86–0.95; *p*-value = 1.86E−05). Inspection of the MTBC phylogeny showed that most strains carrying non-synonymous mutations in *sufD* belonged to two closely related drug-resistant subclades of L2.2.1 “Central Asia,” each one carrying a different mutation: SufD 1650265:T > C/Val247Ala (*n* = 153; 78.4%) and SufD 1650180:C > T/Arg219Trp (*n* = 29; 14.9%), respectively (Fig. 1). The effect of these mutations in reducing the odds of cavitary disease may be linked to drug resistance, as cavitary disease was less common among drug-resistant strains carrying *sufD* mutations (Fig. 1).

### Mutations in *sufD*, MTBC lineage, rifampicin resistance, and compensatory evolution are linked to bacterial burden

We hypothesized that if strains carrying *sufD* mutations are less likely to cause cavitary disease, they should also be linked to lower bacterial burdens. To test this, we modeled the factors that influence TTP (measured in days) as a proxy for bacterial burden (Table 2; *n* = 2105 isolates from pulmonary TB patients with TTP data available; Supplementary Material; Additional File 1: Fig S1). Psychiatric disorders, previous contact with a drug-resistant case, being treated with bedaquiline, delamanid, and/or linezolid, being treated with fluoroquinolones, and mutations conferring higher RIF MICs were associated with a longer TTP (i.e., lower bacillary load), whereas older age, male sex, receiving treatment for other diseases, presenting infiltration (non-cavitary nodular opacities on imaging), dissemination, and cough, as well as being infected with MTBC strains carrying compensatory mutations, were associated with a shorter TTP (i.e., higher bacillary load) (Table 2).

**Table 2:** Factors associated with TTP (time to culture positivity) among all samples with TTP data (*n* = 2105)

**Variable**	**Estimate (days)**	**95% CI** ^=*1*^ ***(days)***	***p*** **-value**
Patient age (years)	−0.02	−0.04, 0.00	**0.018**
Male	−1.0	−1.7, −0.43	**< 0.001**
HIV-coinfection: missing	−0.35	−1.2, 0.51	0.4
HIV-coinfection: positive	1.5	−0.19, 3.3	0.082
Treated with Bdq/Dlm/Lzd	1.0	0.16, 1.9	**0.021**
Treated with fluoroquinolones	1.4	0.17, 2.7	**0.027**
Presence of compensatory mutations	−1.6	−2.5, −0.66	**< 0.001**
Treatment for other diseases: missing	0.35	−1.0, 1.8	0.6
Treatment for other diseases: yes	−1.2	−2.3, −0.04	**0.043**
Drug abuse: missing	−0.04	−1.2, 1.1	> 0.9
Drug abuse: yes	1.7	−0.51, 4.0	0.13
Genotypic RIF MIC	0.26	0.08, 0.44	**0.004**
Genotypic EMB MIC	0.33	−0.05, 0.72	0.091
Cough	−1.7	−3.1, −0.40	**0.011**
Dissemination	−1.5	−2.4, −0.53	**0.002**
Infiltration	−1.1	−2.1, −0.15	**0.024**
Psychiatric disorder	4.1	−0.12, 8.4	0.057
Previous contact with DR case	1.9	0.31, 3.4	**0.019**
Case definition: previous treatment failure	−2.1	−4.6, 0.51	0.12
Case definition: other	0.97	−0.30, 2.2	0.14
Case definition: recurrent TB	0.61	−0.23, 1.5	0.2
Case definition: treatment after defaulting	0.03	−1.3, 1.3	> 0.9
Presence of non-synonymous *sufD* mutations	1.3	−0.13, 2.7	0.076

*Bdq *Bedaquiline, *Dlm* Delamanid,* Lzd *Linezolid

HIV-coinfection (beta = 1.5 days, CI = −0.19–3.3; *p*-value = 0.082) and being infected with strains carrying non-synonymous mutations in *sufD* (beta = 1.3 days, CI = -0.13–2.7; *p*-value = 0.076) also seemed associated with lower bacterial burdens. Since we observed in the phylogeny that the effect of *sufD* may be linked to drug resistance, we repeated the analysis only among drug-resistant cases with available TTP data (*n* = 893). We found that, in addition to the predictors described above, non-synonymous mutations in *sufD* (beta = 1.6 days, CI = 0.16–3.1; *p*-value = 0.03) and MTBC lineage 2 (beta = 1.5 days, CI = 0.49–2.6; *p*-value = 0.004) were associated with a longer TTP (i.e., a lower bacterial burden) among drug-resistant cases; Additional File 1: Table S5; Supplementary Repository).

## Discussion

We analyzed MTBC genomes and associated clinical data from patients diagnosed with TB in Georgia over a 13-year period to identify patient and bacterial determinants of clinical outcomes and disease manifestation. Our results highlight the multifactorial nature of TB disease, confirm the role of known clinical and demographic variables, and support a relevant role of bacterial factors in patient outcomes.

Our work shows that patient factors such as older age, male sex, lower BMI, previous history of TB disease, unemployment, and drug abuse influence treatment outcomes in Georgia. These results align with existing evidence (refer to [5]), and highlight that studies aiming at identifying bacterial factors influencing treatment outcomes need to control for relevant patient covariates. An important aspect of the cohort analyzed here is that a third of all patients with drug resistance were treated with novel drugs (i.e., bedaquiline, delamanid, and/or linezolid). Our results showed a substantial decrease in the odds of unfavorable outcomes when these drugs were used (aOR = 0.22; CI = 0.14–0.37), providing additional evidence of the efficacy of these novel regimens against drug-resistant TB [3].

Among the various bacterial factors that we assessed, enhanced levels of drug resistance and higher bacterial burdens (i.e., a shorter TTP) were associated with unfavorable outcomes (Table 1). Additionally, we found MTBC L2 to be associated with unfavorable outcomes only among drug-susceptible cases, supporting the results from a previous study [11]. We see two potential explanations for these results, which are not mutually exclusive. First, it is possible that L2 strains are overall more virulent, but among drug-resistant isolates, the association is not observed because drug resistance itself has a much stronger effect on treatment outcomes than the MTBC lineage. Second, these results may suggest that L2 strains better survive first-line treatment or are more likely to acquire drug resistance-conferring mutations, therefore leading to treatment failure and relapse in susceptible cases. Although this association has been reported previously, in our study, we only found a weak signal (aOR = 1.51; CI = 1.02–2.19; *p*-value = 0.035), and therefore these results should be interpreted with caution.

Contrary to previous work [23, 24], we did not find mixed infections to be associated with unfavorable outcomes. Similarly, we did not find an association between heteroresistance and unfavorable treatment outcomes. Previous studies have reported contradictory results regarding the association of mixed infections and heteroresistance with treatment outcomes [23–27]. Our results suggest that, although mixed infections and heteroresistance can lead to misdiagnosis and contribute to unfavorable patient outcomes in some cases, as compared to other factors, they likely play a limited role on a broader scale.

An important finding of our study is the individual effects on treatment outcomes of specific bacterial mutations linked to different levels of resistance to fluoroquinolones and rifampicin. These results support the notion that the efficacy of TB drugs does not depend on the bacterial resistance on a strict binary basis, but on the actual level of resistance. As regimens comprising high-dose isoniazid or high-dose rifampicin have been shown to be generally safe [28, 29], our results support the rationale of optimizing treatments for drug-resistant TB with existing drugs.

Beyond drug resistance, we did not find any association between specific MTBC genetic variants and treatment outcomes. These results are in agreement with benchmarks of GWAS methods using MTBC genomic data [30], but contrast with previously published studies performing GWAS, which have reported associations between MTBC genetic variants and treatment outcomes [7, 13–16]. However, these associations were often observed in only a small number of strains, or did not control for relevant clinical variables, and they have not yet been replicated in independent studies. Notably, in our analysis we did not test these variants previously reported in the literature. Future meta-analyses that harmonize methodological differences across studies may help clarify the role of bacterial genetics with treatment outcomes.

By contrast, we found different bacterial features linked to key disease characteristics, including bacterial burden and the presence of lung cavities. Mutations linked to higher MICs to rifampicin were associated with a lower bacterial burden. This observation aligns with previous research showing that drug resistance often imposes a replicative fitness cost on the bacteria [31]. Conversely, compensatory mutations mitigating the fitness cost of rifampicin resistance were associated with higher bacterial burdens, supporting a previous study in which we reported an association between the presence of compensatory mutations and positive smear microscopy in a different setting [32]. Additionally, our study revealed a potential association of non-synonymous mutations in *sufD* with lower bacterial burdens and lower odds of cavitary disease, particularly among drug-resistant cases. These results are compatible with existing evidence from different bacterial species that have shown a critical role of *sufD* in redox metabolism, iron uptake, and bacterial survival [33, 34]. Existing literature also shows an interplay between iron metabolism—and *sufD* in particular—with antibiotic resistance. Iron availability influences resistance to isoniazid, rifampicin, or fluoroquinolones in various bacterial species, including MTBC (reviewed in [35]). Experimental work has shown *sufD* to be upregulated upon rifampicin exposure in *M. smegmatis *[36], as well as in *E. coli* strains carrying *rpoB* mutations [37]. In an experimental evolution setting, two out of 13 *E. coli* strains evolving in media with antibiotics acquired non-synonymous mutations in *sufD *[38]. These results are particularly interesting in the light of a recent study that identified *sufD* as a potential TB drug target [39]. Taken together, these observations suggest that mutations in *sufD* may mediate a trade-off between virulence and antibiotic resistance, highlighting *sufD* as a potential drug target that could prove useful for treating drug-resistant TB. However, while we found potential associations of *sufD* both with the presence of lung cavities and the bacterial burden within hosts, the relatively small effect size and weak statistical signal (*p*-values of 1.86E−5 and 0.024 for the GWAS [cavitary disease] and logistic regression [TTP], respectively) call for a cautious interpretation of these results. Future studies that use targeted functional genomics, in vivo infection models, as well as other studies further testing the association between *sufD*, bacterial growth, and disease severity, will help clarify the role of this gene in mediating virulence and drug resistance.

Our study has several limitations. First, we modeled drug MICs based on specific mutations, but it is known that MTBC strains carrying the same drug resistance-conferring mutations can show differences in MIC for the same drug [9]. However, the estimated effect of the mutations on the MICs were determined based on two different studies that modeled a large dataset of phenotypic and genotypic data derived from the CryPTIC consortium [6, 19]. Additionally, the MTBC samples analyzed here also included more than 1300 drug-resistant strains from a single country, with relatively low genetic diversity, which together should minimize potential biases in this analysis. An additional limitation of our analysis is that the effect of isoniazid and pyrazinamid resistance could not be modeled, since isoniazid resistance was dominated by a single mutation (KatG Ser315Thr, Additional File 1: Fig S2), and MIC values for pyrazinamide were not available. Second, the relatively low genetic diversity of the MTBC strains circulating in Georgia, most of which belong to L2 and L4, may have precluded finding further associations between bacterial genetics and TB treatment outcomes. Our GWAS also presents limitations. First, we focus our analysis on detecting clinically relevant associations of bacterial genetic traits with treatment outcomes. Therefore, we adjusted our analyses by bacterial population structure and clinical covariates. While this conservative approach reduces the risk of reporting spurious associations, it inherently comes at the cost of reduced sensitivity, and thus, we may have missed weaker yet biologically meaningful associations. Additionally, in the GWAS, we only investigated SNPs and small indels, but larger structural variants may confer phenotypes relevant to treatment outcomes [13].

## Conclusions

In conclusion, our combined analysis of patient and bacterial variables from a large cohort of TB patients in Georgia over a 13-year period highlighted key demographic, clinical, and bacterial determinants of TB treatment outcomes. In addition to confirming known patient factors, we showed that the MTBC lineage and specific drug resistance-conferring mutations were associated with treatment outcomes, and that additional bacterial variables such as the presence of compensatory mutations and mutations in *sufD* can influence disease presentation. Our results underscore the need to consider both patient and bacterial factors when optimizing TB treatment regimens.

## Supplementary Information

Additional File 1: Supplementary Methods, Results, Figures and Tables.

## Data Availability

The full dataset with deidentified patient- and bacterial-related data analyzed in this study is available in a public repository in Zenodo [21]. The repository also contains a detailed description of every analysis performed in R-markdown, that allows full reproducibility of the results, along with supplementary results, tables and figures. Raw sequencing data for the analyzed MTB isolates were deposited in the European Nucleotide Archive under the study projects PRJEB39561, PRJEB50582 and PRJEB74234, with a corresponding list of sample accession numbers available in the primary repository.

## Abbreviations

aOR: Adjusted odds ratio
Bdq: Bedaquiline
BPaL(M): Bedaquiline, pretomanid, linezolid (with or without moxifloxacin)
BMI: Body mass index
CI: Confidence interval
Dlm: Delamanid
DR: Drug-resistant
EMB: Ethambutol
GWAS: Genome-wide association studies
HIV: Human immunodeficiency virus
INH: Isoniazid
LASSO: Least absolute shrinkage and selection operator
Lzd: Linezolid
MDR-TB: Multidrug-resistant tuberculosis
MIC: Minimum inhibitory concentration
MTBC: *Mycobacterium tuberculosis* complex
pre-XDR: Pre-extensively drug-resistant
RIF: Rifampicin
RR: Rifampicin-resistant
SNP: Single nucleotide polymorphism
TB: Tuberculosis
TTP: Time to culture positivity
WHO: World Health Organization
WGS: Whole-genome sequencing
XDR-TB: Extensively drug-resistant tuberculosis
