# Supplementary material for: Clinical and bacterial determinants of unfavorable tuberculosis treatment outcomes: an observational study in Georgia
Source: Genome Med. 2025 Nov 14;17:143. doi: 10.1186/s13073-025-01555-0 (PMC12619330; doi:10.1186/s13073-025-01555-0)
Supplement: Supplementary file 1 — Additional File 1: Supplementary Methods, Results, Figures and Tables. [file 13073_2025_1555_MOESM1_ESM.docx]

# Supplementary Material

## **Clinical and bacterial determinants of unfavorable tuberculosis treatment outcomes: an observational study in Georgia.**

Galo A. Goig^1,2✉^, Chloé Loiseau^1,2^, Nino Maghradze^1,2,3^, Kakha Mchedlishvili^1,2,3^, Teona Avaliani^3^, Ana Tsutsunava^3^, Daniela Brites^1,2^, Sevda Kalkan^1,2^, Sonia Borrell^1,2^, Rusudan Aspindzelashvili^3^, Zaza Avaliani^3,4^, Maia Kipiani^3,5,6^, Nestani Tukvadze^1,2,3^, Levan Jugheli^1,2,3^ and Sebastien Gagneux^1,2^

1 - Swiss Tropical and Public Health Institute, Allschwil, Switzerland.

2 - University of Basel, Basel, Switzerland.

3 - National Center for Tuberculosis and Lung Diseases (NCTLD), Tbilisi, Georgia.

4 - European University, Tbilisi, Georgia.

5 - David Tvildiani Medical University (DTMU), Tbilisi, Georgia.

6 - The University of Georgia, Tbilisi, Georgia.

All data and a detailed step-by-step description of all analyses performed in this study are available in a public Zenodo repository[[1]](https://paperpile.com/c/i9dENk/b5hE). The R scripts in the repository allow to reproduce the results shown in the study, as well as to explore and reuse the data. Additionally, we provide in this file the main supplementary methods and results to facilitate the readability and understanding of the main text.

**Supplementary Methods**

**Selection of predictors of unfavorable tuberculosis treatment outcomes**

To select the most relevant predictors of unfavorable tuberculosis treatment outcomes among the variables considered in this study (Supplementary Repository), we first built a multivariable logistic regression with LASSO regularization using the value of lambda with the highest AUC (Supplementary Results), and initially included any predictor with non-zero coefficient. Since some of the predictors could be collinear, we additionally built a random forest with 1000 trees (6 variables per tree) to include any predictor ranking among the top ten most relevant (measured as the mean decreased Gini index) that were not included according to the LASSO regression. Year of diagnosis, patient age, patient sex and HIV coinfection were always included as predictors, regardless of the results of the LASSO and random forest regressions. Finally, a 10-fold cross-validated multivariable logistic regression model with treatment outcome as binary outcome was built using the predictors selected using this approach. To simplify this initial “base model”, we removed predictors with a p-value > 0.2, and we also excluded specific drug resistance-conferring mutations, *Mycobacterium tuberculosis* (MTB) complex lineages/sublineages, heteroresistance, or mixed infections, since all these predictors were further evaluated in specific analyses.

**Modeling the effect of the MIC imputed to specific drug resistance-conferring mutations on tuberculosis treatment outcomes**

In this study we sought to model the effect that specific drug resistance-conferring mutations have on tuberculosis treatment outcomes through their effect on the minimum inhibitory concentration (MIC). Our study focused on modeling the effect of resistance to rifampicin and fluoroquinolones, since resistance to these drugs emerged as relevant predictors of unfavorable outcomes (Table 1; Supplementary Repository), and resistance to isoniazid and pyrazinamide could not be modeled as explained in the main text. Each MTB isolate was assigned two sets of rifampicin and fluoroquinolone MICs, one using the effect of drug resistance-conferring mutations on the MIC as estimated by Barilar et al. [[2]](https://paperpile.com/c/i9dENk/fKFlE), and another one using the effect on the MIC as estimated by Wiatrak et al. [[3]](https://paperpile.com/c/i9dENk/Z7B77). In both cases, the effect of each mutation on the MIC are detailed in the supplementary material of the respective studies. To test the effect that the estimated MICs have on unfavorable treatment outcomes of tuberculosis, we first substituted in the base model the drug resistance profile by the estimated MIC to rifampicin, and adjusted the analysis by fluoroquinolone resistance (encoded as fluoroquinolone resistant/susceptible). This analysis was performed only for isolates with rifampicin resistance mutations, as no MIC value can be imputed otherwise based on genomic analysis (n=1,348). Afterwards, we substituted in the model fluoroquinolone resistance (previously encoded as resistant/susceptible), by the estimated fluoroquinolone MIC. This analysis was performed only for isolates with both rifampicin and fluoroquinolone resistance mutations (n=344). These analyses were performed twice, one for each set of estimated effects on the MIC (Tables S2 and S3).

**Supplementary Results**

**Predictors of unfavorable tuberculosis treatment outcomes**

The value of lambda that maximized the AUC in the LASSO regression was 0.005341, and the predictors with non-zero coefficients according to the LASSO regularization are shown in Fig S3. The top ten most relevant predictors based on a random forest were, in order of decreasing relevance: patient age, BMI, time to culture positivity (TTP), year of diagnosis, case definition, ratio of unfixed to fixed variants (HH ratio), MTBC sublineage, drug resistance profile, sex, dyspnea, HIV and imprisonment. After simplifying the initial “base model”, we excluded HH ratio (p-value = 0.8) and imprisonment (p-value=0.7) as predictors (Table 1, Supplementary Repository). The final predictors of the base model and their corresponding coefficients, 95% confidence intervals, and p-values are shown in Table 1.

**Supplementary Figures**

Fig S1 - Study flowchart showing inclusion and exclusion criteria, along with the number of samples considered in each analysis. Abbreviations: MTB (*Mycobacterium tuberculosis*), TB (Tuberculosis), RR/MDR (Rifampicin-resistant/Multidrug-resistant), WGS (Whole-genome sequencing), TTP (Time to culture positivity), EPTB (Extrapulmonary tuberculosis). WGS data was considered high-quality if the average sequencing depth was higher than 20-fold and the percentage of reads classified by Kraken[[4]](https://paperpile.com/c/i9dENk/1KaGP) as MTB was higher than 90%. In this flowchart, isoniazid monoresistant cases (n=70) are categorized as susceptible TB. For each TB patient, we analyzed the last MTB isolate available from the last TB episode. To analyze factors associated with TTP, we only considered cases of pulmonary TB.


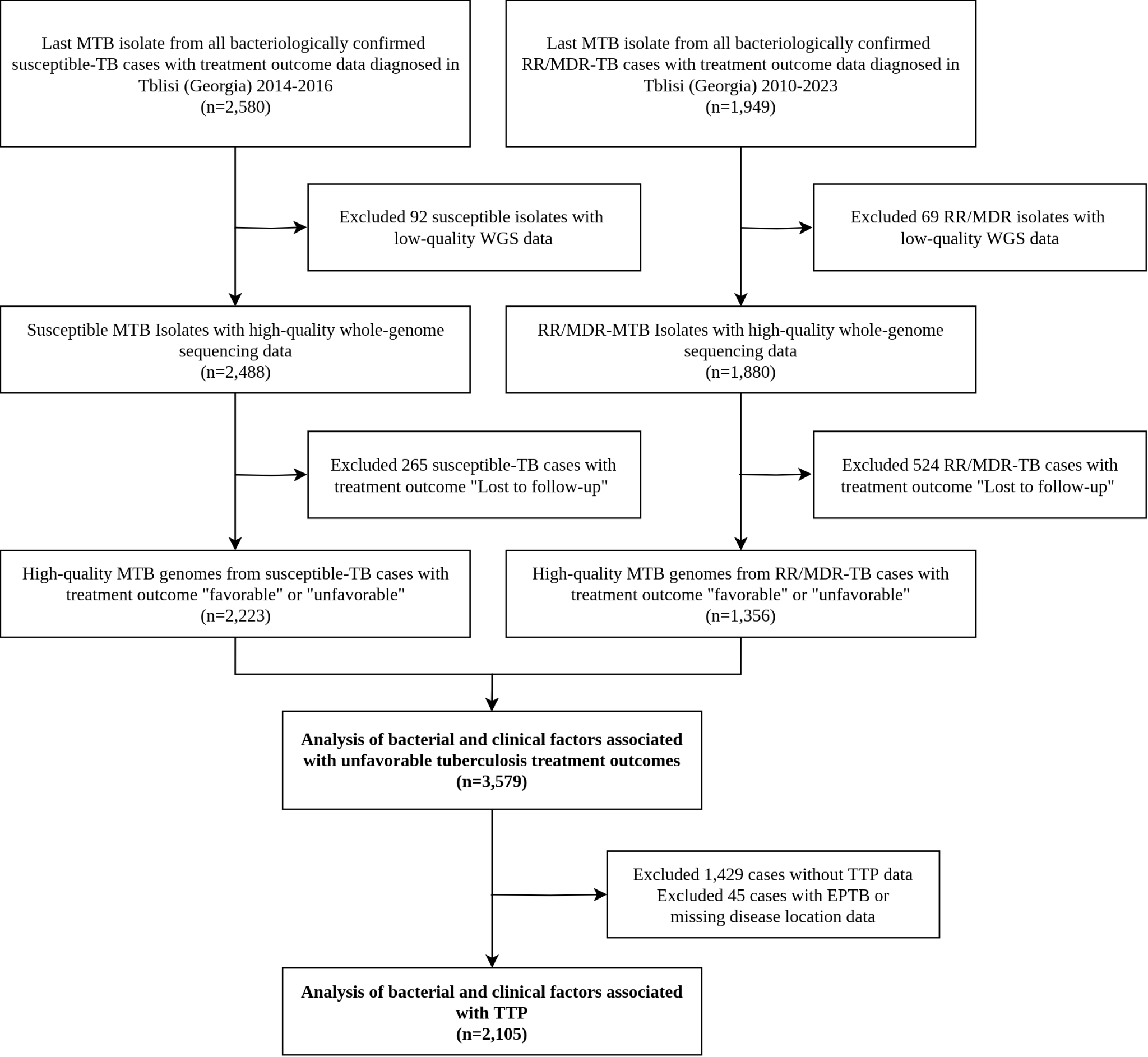


Fig S2 - Distribution of drug resistance-conferring mutations to ethambutol (EMB), fluoroquinolones (FQ), isoniazid (INH), pyrazinamide (PZA) and rifampicin (RIF). The category “Other” contains mutations with less than 20 observations. The category “Mixed” corresponds to observations of different drug resistance-conferring mutations to the same drug in the same isolate.


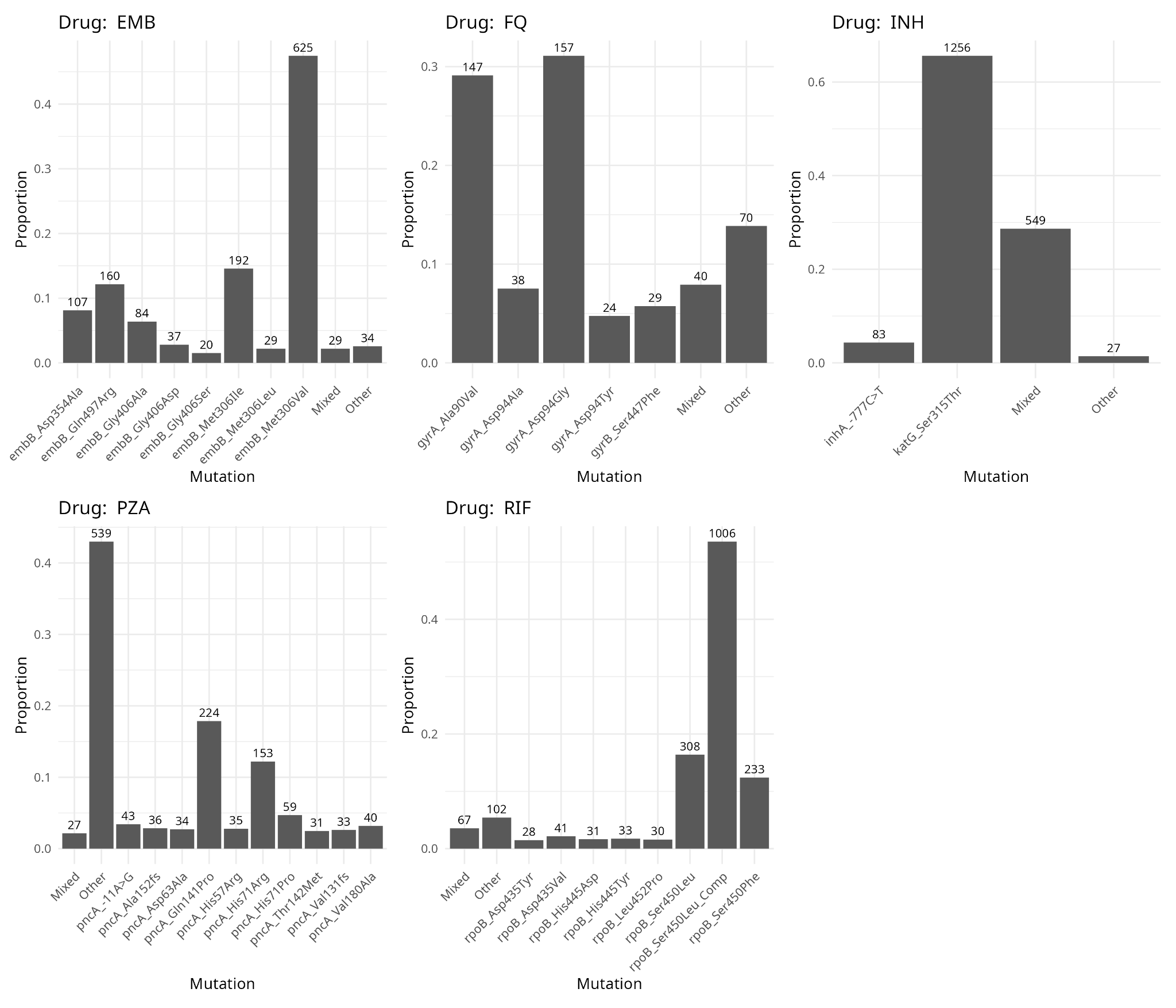


Fig S3 - Predictors with non-zero coefficients after LASSO regularization. The horizontal bars represent the LASSO coefficients for lambda = 0.005341. Coefficients were coloured in light red to note increased odds of unfavorable outcomes, and in light blue to note decreased odds of unfavorable outcomes. Abbreviations: XDR (Extensively drug-resistant), TB (tuberculosis), BDQ/DLM/LZD (Bedaquiline, Delamanid, Linezolid), MTBC (*Mycobacterium tuberculosis* complex).

**
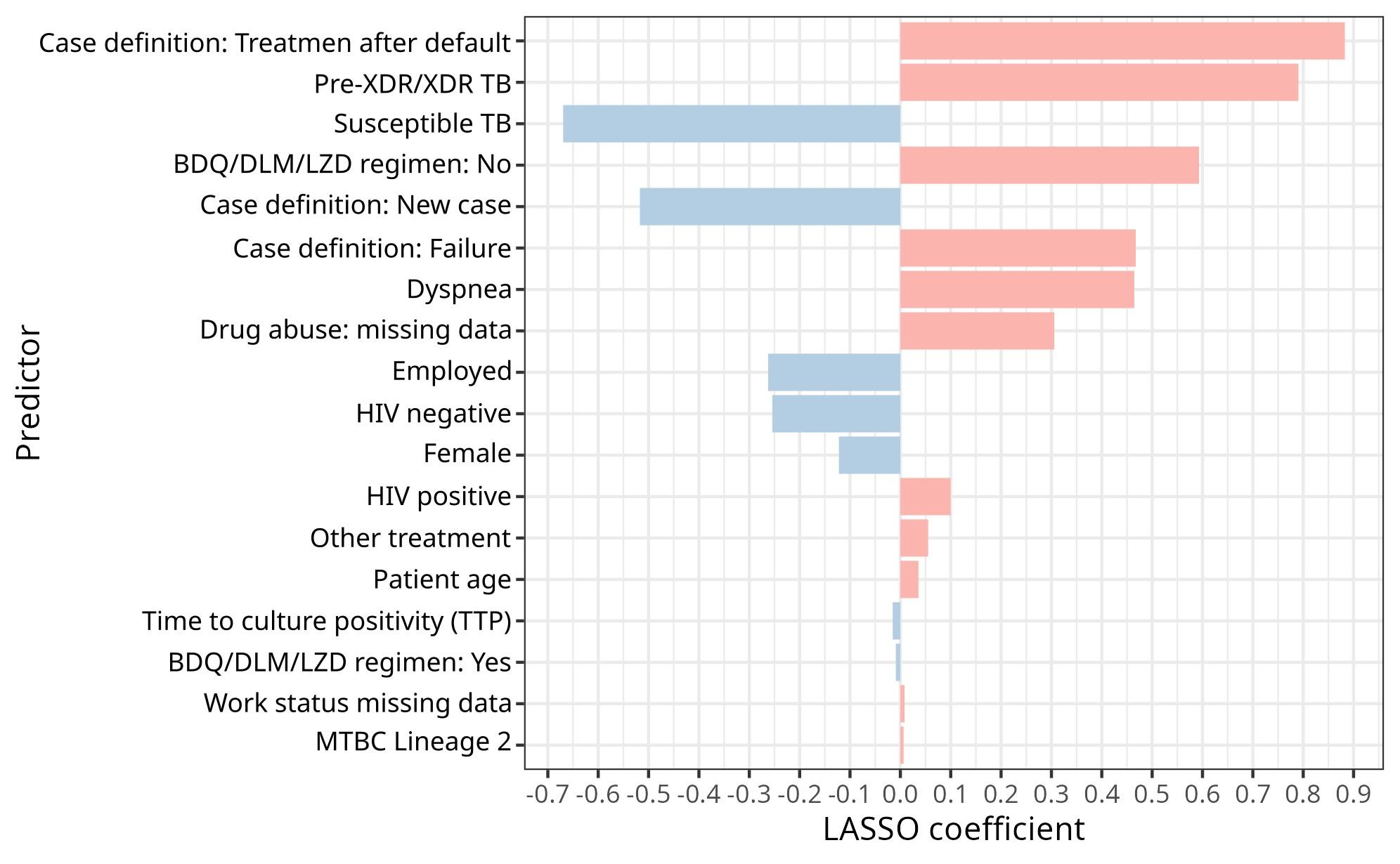
**

**Supplementary Tables**

Table S1 - Multivariable logistic regression of factors associated with unfavorable treatment outcomes among drug-susceptible TB cases (n=2,153, excluding isoniazid monoresistant TB).

| **Variable** | **Odds ratio** | **95% CI (Odds ratio)** | **p-value** |
| --- | --- | --- | --- |
| Lineage 2 | 1.51 | 1.02–2.19 | **0.035** |
| Mixed infection (lineage) | 2.81 | 0.42–11.0 | 0.2 |
| Uncommon lineage | 1.96 | 0.10–10.9 | 0.5 |
| Year of diagnosis | 0.98 | 0.89–1.08 | 0.7 |
| Age (years) | 1.06 | 1.04–1.07 | **<0.001** |
| Male sex | 1.80 | 1.16–2.85 | **0.010** |
| Work status missing | 2.29 | 0.85–6.01 | 0.093 |
| Unemployed | 1.26 | 0.70–2.50 | 0.5 |
| Case definition: Failure | 4.41 | 1.00–17.1 | **0.036** |
| Case definition: Other | 1.38 | 0.58–2.88 | 0.4 |
| Case definition: Recurrent tuberculosis | 1.67 | 0.95–2.79 | 0.061 |
| Case definition: Treatment after default | 3.05 | 1.25–6.68 | **0.008** |
| HIV status missing | 1.15 | 0.75–1.74 | 0.5 |
| HIV positive | 1.04 | 0.16–3.78 | >0.9 |
| Body Mass Index (kg/m2) | 1.04 | 0.94–1.16 | 0.4 |
| Time to Culture Positivity (days) | 0.93 | 0.89–0.97 | **0.001** |

Table S2 - Multivariable logistic regression of factors associated with unfavorable treatment outcomes, substituting the drug resistance profile with rifampicin and fluoroquinolone MICs, as estimated by Barilar et al. [[2]](https://paperpile.com/c/i9dENk/fKFlE). In this analysis, the MIC units are log_2_-based, and therefore the effect on the odds of unfavorable outcomes corresponds to each doubling of the MIC value. Analysis of isolates presenting both rifampicin and fluoroquinolone resistance-conferring mutations (n=344). Abbreviations: TB (tuberculosis); BDQ/DLM/LZD (Bedaquiline, Delamanid, Linezolid).

| **Variable** | **Odds Ratio (OR)** | **95% CI (Odds ratio)** | **p-value** |
| --- | --- | --- | --- |
| Year of Diagnosis | 1.04 | 0.99, 1.10 | 0.14 |
| Age (years) | 1.05 | 1.04, 1.06 | **<0.001** |
| Male Sex | 1.65 | 1.22, 2.26 | **0.002** |
| HIV Status Missing | 1.45 | 1.04, 2.00 | **0.026** |
| HIV Positive | 1.77 | 0.98, 3.13 | 0.053 |
| Body Mass Index (kg/m2) | 0.95 | 0.91, 1.00 | **0.039** |
| Time to Culture Positivity (days) | 0.95 | 0.93, 0.98 | **<0.001** |
| Case Definition: Failure | 5.32 | 2.52, 11.1 | **<0.001** |
| Case Definition: Other | 1.47 | 0.92, 2.30 | 0.10 |
| Case Definition: Recurrent TB | 1.79 | 1.26, 2.51 | **<0.001** |
| Case Definition: Treatment After Default | 4.92 | 3.21, 7.49 | **<0.001** |
| Work Status Missing | 3.37 | 1.79, 6.47 | **<0.001** |
| Unemployed | 2.14 | 1.34, 3.58 | **0.002** |
| Drug Use Status Missing | 2.95 | 1.90, 4.56 | **<0.001** |
| Drug Use: Yes | 0.60 | 0.17, 1.70 | 0.4 |
| Other Treatment Info Missing | 0.87 | 0.47, 1.56 | 0.6 |
| Other Treatment: Yes | 1.61 | 1.04, 2.48 | **0.033** |
| Dyspnea Info Missing | 2.32 | 1.11, 4.77 | **0.023** |
| Dyspnea: Yes | 2.41 | 1.54, 3.72 | **<0.001** |
| Bdq/Dlm/Lzd Regimen: Yes | 0.21 | 0.13, 0.35 | **<0.001** |
| Fluoroquinolone log_2_(MIC) | 1.36 | 1.23, 1.51 | **<0.001** |
| Rifampicin log_2_(MIC) | 1.28 | 1.20, 1.36 | **<0.001** |

Table S3 - Multivariable logistic regression of factors associated with unfavorable treatment outcomes, substituting the drug resistance profile with rifampicin and fluoroquinolone MICs, as estimated by Wiatrak et al. [[3]](https://paperpile.com/c/i9dENk/Z7B77). Analysis of isolates presenting both rifampicin and fluoroquinolone resistance-conferring mutations (n=344). Abbreviations: TB (tuberculosis); BDQ/DLM/LZD (Bedaquiline, Delamanid, Linezolid).

| **Variable** | **Odds Ratio (OR)** | **95% CI (Odds ratio)** | **p-value** |
| --- | --- | --- | --- |
| Year of Diagnosis | 1.04 | 0.99, 1.10 | 0.13 |
| Age (years) | 1.05 | 1.04, 1.06 | **<0.001** |
| Male Sex | 1.65 | 1.22, 2.26 | **0.001** |
| HIV Status Missing | 1.46 | 1.05, 2.02 | **0.021** |
| HIV Positive | 1.75 | 0.97, 3.08 | 0.058 |
| Body Mass Index (kg/m2) | 0.95 | 0.91, 1.00 | **0.036** |
| Time to Culture Positivity (days) | 0.95 | 0.93, 0.98 | **<0.001** |
| Case Definition: Failure | 5.41 | 2.57, 11.2 | **<0.001** |
| Case Definition: Other | 1.49 | 0.94, 2.32 | 0.085 |
| Case Definition: Recurrent TB | 1.83 | 1.29, 2.56 | **<0.001** |
| Case Definition: Treatment After Default | 5.09 | 3.33, 7.74 | **<0.001** |
| Work Status Missing | 3.31 | 1.76, 6.32 | **<0.001** |
| Unemployed | 2.09 | 1.32, 3.49 | **0.003** |
| Drug Use Status Missing | 2.94 | 1.89, 4.55 | **<0.001** |
| Drug Use: Yes | 0.59 | 0.17, 1.69 | 0.4 |
| Other Treatment Info Missing | 0.82 | 0.44, 1.48 | 0.5 |
| Other Treatment: Yes | 1.56 | 1.01, 2.40 | **0.046** |
| Dyspnea Info Missing | 2.01 | 0.96, 4.19 | **0.063** |
| Dyspnea: Yes | 2.37 | 1.52, 3.67 | **<0.001** |
| Bdq/Dlm/Lzd Regimen: Yes | 0.22 | 0.13, 0.36 | **<0.001** |
| Fluoroquinolone MIC | 2.94 | 1.97, 4.38 | **<0.001** |
| Rifampicin MIC | 3.73 | 2.61, 5.38 | **<0.001** |

Table S4 - Multivariable logistic regression of factors associated with unfavorable treatment outcomes, including heteroresistance to isoniazid and fluoroquinolones. In univariate analysis, heteroresistance to isoniazid and fluoroquinolones showed to be potentially associated with treatment outcomes (Supplementary Repository). Abbreviations: TB (tuberculosis); INH (isoniazid); RR/MDR (Rifampicin-resistant/Multidrug-resistant); XDR (extensively drug-resistant); BDQ/DLM/LZD (Bedaquiline, Delamanid, Linezolid); FQ (fluoroquinolone).

| **Variable** | **Odds Ratio (OR)** | **95% CI (Odds ratio)** | **p-value** |
| --- | --- | --- | --- |
| Year of Diagnosis | 1.04 | 0.99, 1.10 | 0.14 |
| Age (years) | 1.05 | 1.04, 1.06 | **<0.001** |
| Male Sex | 1.66 | 1.22, 2.27 | **0.001** |
| HIV Status Missing | 1.50 | 1.07, 2.07 | **0.016** |
| HIV Positive | 1.69 | 0.93, 2.99 | 0.079 |
| Body Mass Index (kg/m2) | 0.95 | 0.91, 0.99 | **0.027** |
| Time to Culture Positivity (days) | 0.95 | 0.92, 0.97 | **<0.001** |
| Case Definition: Failure | 4.51 | 2.10, 9.52 | **<0.001** |
| Case Definition: Other | 1.44 | 0.90, 2.26 | 0.12 |
| Case Definition: Recurrent TB | 1.84 | 1.30, 2.58 | **<0.001** |
| Case Definition: Treatment After Default | 4.68 | 3.04, 7.17 | **<0.001** |
| INH-Monoresistant TB | 3.86 | 1.61, 8.47 | **0.001** |
| RR/MDR-TB | 8.81 | 5.16, 15.1 | **<0.001** |
| Pre-XDR/XDR-TB | 23.2 | 12.8, 42.9 | **<0.001** |
| Work Status Missing | 3.41 | 1.80, 6.61 | **<0.001** |
| Unemployed | 2.19 | 1.36, 3.71 | **0.002** |
| Drug Use Status Missing | 2.99 | 1.94, 4.60 | **<0.001** |
| Drug Use: Yes | 0.64 | 0.19, 1.82 | 0.4 |
| Other Treatment Info Missing | 0.85 | 0.46, 1.51 | 0.6 |
| Other Treatment: Yes | 1.69 | 1.09, 2.60 | **0.017** |
| Dyspnea Info Missing | 2.64 | 1.28, 5.36 | **0.008** |
| Dyspnea: Yes | 2.33 | 1.50, 3.60 | **<0.001** |
| Bdq/Dlm/Lzd Regimen: Yes | 0.23 | 0.14, 0.38 | **<0.001** |
| FQ Heteroresistance: Yes | 1.70 | 0.74, 3.85 | 0.2 |
| INH Heteroresistance: Yes | 0.24 | 0.04, 0.91 | 0.070 |

Table S5 - Multivariable linear regression of factors associated with time to culture positivity (TTP) among drug-resistant pulmonary TB cases with available TTP data (n=893). Abbreviations: BDQ/DLM/LZD (Bedaquiline, Delamanid, Linezolid); FQ (Fluoroquinolones); TB (tuberculosis); MTBC (*Mycobacterium tuberculosis* complex); MIC (Minimum inhibitory concentration); DR-TB (drug-resistant tuberculosis).

| **Variable** | **Coefficient (days)** | **95% Confidence Interval** | **p-value** |
| --- | --- | --- | --- |
| Male Sex | -0.79 | -1.8, 0.24 | 0.13 |
| Bdq/Dlm/Lzd Regimen: Yes | 0.91 | 0.07, 1.7 | **0.033** |
| FQ Regimen: Yes | 1.2 | 0.00, 2.4 | **0.051** |
| Lineage 2 | 1.5 | 0.49, 2.6 | **0.004** |
| Presence of compensatory mutations | -1.9 | -2.7, -1.0 | **<0.001** |
| Other Treatment Info Missing | 0.39 | -0.96, 1.7 | 0.6 |
| Other Treatment: Yes | -1.3 | -2.4, -0.25 | **0.016** |
| Drug Use Status Missing | 0.22 | -0.90, 1.4 | 0.7 |
| Drug Use: Yes | 1.9 | -0.26, 4.0 | 0.086 |
| Alcohol Intake: Excessive | -1.4 | -3.0, 0.23 | 0.092 |
| Alcohol Intake: Moderate | -0.50 | -1.5, 0.49 | 0.3 |
| Rifampicin MIC (per MIC unit increase) | 0.26 | 0.04, 0.47 | **0.019** |
| Cough: Yes | -1.8 | -3.1, -0.46 | **0.008** |
| Disseminated TB | -1.5 | -2.4, -0.59 | **0.001** |
| Radiological Infiltrate | -1.2 | -2.1, -0.26 | **0.013** |
| Psychiatric Condition: Yes | 5.4 | 1.2, 9.7 | **0.012** |
| Contact with DR-TB Case: Yes | 2.0 | 0.52, 3.5 | **0.008** |
| Case Definition: Failure | -1.6 | -4.3, 1.2 | 0.3 |
| Case Definition: Other | 1.8 | 0.22, 3.3 | **0.025** |
| Case Definition: Recurrent TB | 0.74 | -0.33, 1.8 | 0.2 |
| Case Definition: Treatment After Default | -0.59 | -2.1, 0.90 | 0.4 |
| Presence of non-synonymous *sufD* mutations | 1.6 | 0.16, 3.1 | **0.030** |

**References**

[1. Goig G, Loiseau C. Clinical and bacterial determinants of unfavorable tuberculosis treatment outcomes: an observational study in Georgia. [cited 2025 Sep 24]; Available from:](http://paperpile.com/b/i9dENk/b5hE) <https://zenodo.org/records/17171544>

[2. The CRyPTIC Consortium. Quantitative measurement of antibiotic resistance in Mycobacterium tuberculosis reveals genetic determinants of resistance and susceptibility in a target gene approach. Nat Commun. 2024;15:1–13.](http://paperpile.com/b/i9dENk/fKFlE)

[3. Wiatrak M, Weimann A, Dinan A, Brbić M, Floto RA. Sequence-based modelling of bacterial genomes enables accurate antibiotic resistance prediction [Internet]. bioRxiv. 2024. Available from:](http://paperpile.com/b/i9dENk/Z7B77) <http://biorxiv.org/lookup/doi/10.1101/2024.01.03.574022>

[4. Wood DE, Salzberg SL. Kraken: ultrafast metagenomic sequence classification using exact alignments. Genome Biology. 2014;15:1–12.](http://paperpile.com/b/i9dENk/1KaGP)
